# Kinetics of Ehrlich ascites tumor growth within millipore chambers in normal and immunized mice.

**DOI:** 10.1038/bjc.1968.8

**Published:** 1968-03

**Authors:** M. Brenneman, P. G. Rigby


### KINETICS OF EHRLICH ASCITES TUMOR GROWTH WITHIN MILLIPORE CHAMBERS IN NORMAL AND IMMUNIZED MICE

M. BRENNEMAN AND P. G. RIGBY

*From the Eppley Institute for Research in Cancer and Allied Diseases, University of Nebraska College of Medicine, 42nd and Dewey, Omaha, Nebraska, U.S.A.*

Received for publication September 28, 1967

THE proliferation of malignant cells has been shown to depend not only on intrinsic neoplastic properties but also on environmental circumstances, *in vivo* (host) or *in vitro* (medium). In addition, a changing rate of growth in a continuing environment has been documented in tumor-bearing hosts and tissue culture situations. The kinetics of neoplastic cells (i.e. Ehrlich ascites tumor cells—EAT) have been studied with regard to cellular proliferation within cell-tight millipore chambers by Amos and Wakefield (1958) (among others), particularly concerning the growth rate of cells in normal mice and the rate of entry of iso-antibody into the chambers. The present investigations define the growth pattern of Ehrlich ascites tumor cells within the restricted environment of millipore chambers in actively immunized mice.

### MATERIALS AND METHODS

White Swiss mice of both sexes (HA/1CR strain), 3–4 months old, were used in this study. The tumor cells used were Lettré strain, hyperdiploid Ehrlich ascites tumor originally obtained from Roswell Park Memorial Institute and grown locally through passage in HA/1CR mice. Millipore chambers, 14 mm. × 2 mm., were used. The rings were No. PR0001401, with filling holes, and the filters used were No. HAWP01400, with a porosity of 0·45 microns. The chambers were assembled using MF cement No. 1. Fresh peritoneal fluid from an 8-day tumor-bearing mouse was harvested and cell counts obtained. A sterile preparation of ten million cells per cu. ml. in normal saline was then prepared. One-tenth ml. of this suspension (one million cells) was then injected into the chamber and the hole plugged by special nylon thread. The chambers were then implanted intraperitoneally in the designated mice.

Mice were anesthetized using 10% Dibutal in doses of 0·25 ml. to 0·4 ml. A depilatory agent (Nair) was used to remove hair, and a $\frac{3}{4}$-inch incision was made using sterile precautions in the lower right quadrant of the abdomen for placement of the chamber intraperitoneally. The wound was closed with two 9 mm. metal clips and Neomycin powder applied. The metal clips were removed from the mice 2 days post-operatively.

At specific times, the chambers were withdrawn and thoroughly washed in saline to remove all tissue, cells, and debris on the outside. The filters were then freed from the ring, and all parts were dropped into a 50 ml. sterile tube containing 2 ml. of normal saline. The tube and the contents were vigorously agitated on a Vortex junior mixer for 5 minutes to break up tumor clumps and suspend all cells equally in the saline. The suspension was filtered through a small piece of glass wool to remove filter particles. Cell counts were performed on this suspension using a hemocytometer and the total number of cells contained in the chamber calculated.

Antigen for immunization was obtained from the peritoneal cavity of tumor-bearing mice. Peritoneal fluid was centrifuged in a graduated tube to note packed cell volume. The supernatant was discarded and the cells resuspended in an equal volume of 0·85% saline. This suspension was then dispersed in 2 ml. aliquots for lyophilization and storage (each tube then contained the dried constituents of 1 ml. of tumor cells). Before injection, 1 ml. of 0·85% saline and 1 ml. of Freund's complete adjuvant were added to this dried powder and the components thoroughly mixed for 10 minutes on a Vortex junior mixer. The following schedule for injectons was used:

Day 1—0·25 ml. antigen intramuscularly (i.m.) (rear legs)
Day 8—0·30 ml. antigen intraperitoneally (i.p.)
Day 15—0·30 ml. antigen i.p.
Day 22—0·30 ml. antigen i.p.
Day 29—0·30 ml. antigen i.p.

The weight of 1 ml. of dry tumor cells was 36 mg. Therefore, 36 mg. of cell fragments was dissolved in 1 ml. of normal saline and 1 ml. of Freund's adjuvant (giving approximately 26 mg. of cell fragments per mouse over the 5-week immunization period).

### RESULTS

### 1. *EAT growth curve in chambers in normal mice* (*Fig.* 1)

Forty-one mice were used in deriving this normal growth curve. Readings were taken at 2-day intervals using at least 2 mice for each determination. When changes in rate were taking place rapidly, larger numbers of mice were used in determining cell counts of chambers to ensure accuracy. The growth " curve " represents a smooth curve drawn between points representing average cell counts found at specified times.

Tumor cell growth was first characterized by an exponential increase in the number of cells, with little if any initial lag in this system. Beginning with $1 \times 10^6$ cells per chamber, there was an increase to $6 \times 10^6$ cells. This initial phase was followed by a period in which the total cell counts per chamber approached an asymptote with approximately $8{\cdot}5 \times 10^6$ cells per chamber.

### 2. *EAT growth rate in chambers in immune mice* (*Fig.* 1)

Growth rate curves in immune mice were determined in the same manner as in normal mice, beginning with $1 \times 10^6$ cells in the chambers and sampling at 2-day intervals. In Fig. 1, it can be seen that there was similar proliferation up to the fourth day. After this time, varying amounts of cell destruction were observed. This 4-day period of similar growth may represent the time needed for equilibration of humoral cytotoxic substances between the peritoneal fluid and the chamber. It appears that the longer the mouse had been immune before receiving the chamber with EAT, the greater the amount of cell destruction seen after the 4-day period. The EAT cells were not completely eliminated by the immune mice within the chambers at any of the time intervals. Table I is a composite chart showing the average normal count plus an average of the counts determined in the immune mice related to the interval after immunization.

FIG. 1.—Ehrlich ascites tumor (EAT) growth in millipore chambers in normal and immunized mice. The heavy dark line is the growth rate curve of EAT in millipore chambers in normal mice. The light straight lines (★ ■ ●) show an " average " of cell counts of EAT in chambers in immune mice. The numbers underneath the lines indicate the number of mice sacrificed for chamber count on a specific day. The three groups of immune mice (★ ■ ●) vary in the interval post-immunization prior to implantation of chambers, as detailed in Table 2. (★—7 days, ■—21 days, ●—180 days.)

TABLE I.—*The Relationship Between the Interval After Immunization and the Average Number of EAT Cells in the Millipore Chamber (see Fig.* 1)

| Experiment symbol | Number of mice | Number of days post-immunization before implantation of EAT in chambers | Average of the total number of all counts of EAT cells in the chambers after 4 days implantation |
|---|---|---|---|
| ——— | 41 | Not immunized, normal control | $5{\cdot}1 \times 10^6$ |
| —★— | 20 | 7 days | $2{\cdot}42 \times 10^6$ |
| - - ■ - - | 17 | 21 days | $1{\cdot}91 \times 10^6$ |
| - - - - ● - - - - | 13 | 180 days | $1{\cdot}36 \times 10^6$ |

3. *The growth of EAT intraperitoneally in normal and immune mice*

To correlate the " chamber " studies and the normal processes of EAT growth in normal mice and mice immunized by the present method, two groups of animals were employed. Twenty-three normal mice (11 female, 12 male) and 28 immune mice (14 female, 14 male) were injected with $1 \times 10^6$ EAT 21 days after the immunization procedure was completed.

The death of these mice after intraperitoneal EAT injection is recorded in Table II. It is apparent that the normal mice died at $21 \pm 2$ days, whereas

TABLE II.—*Death After EAT Intraperitoneally* (Days)

| Mice | Number | 1–19 | 20–22 | 23–30 | 30–40 | 40–60 | 60–90 | 90+ |
|---|---|---|---|---|---|---|---|---|
| Normal | 23 | 0 | 18 | 3 | 1 | 1 | 0 | 0 |
| Immune | 28 | 0 | 2 | 1 | 0 | 5 | 1 | 17 |

immunized mice receiving the same dose of EAT had a definitely prolonged longevity. It is also apparent from these two groups of mice, as well as the chamber studies, that there is individual variation in the response from mouse to mouse. It was noted that approximately 1 in 15 mice showed an apparent natural resistance to EAT; after prolonged retention and observation, these mice eventually died of the tumor between 40 and 60 days. In the immunized group, a similar ratio of mice was noted which did not develop an immunity to EAT and died at the same time as normal mice treated similarly.

4. *Control studies on cell counting evaluation*

To check for cell loss during the counting procedure outlined above, the ring and filters were resuspended in 2 ml. of saline and thoroughly agitated. Additional cell counts were done on the supernatant. The glass wool filter was treated by vigorous agitation, suspended in saline, and cell counts done on this supernatant also. These controls produced negligible cell counts, thereby indicating that the methods used accounted for approximately 97% of the cells. Therefore, few cells (approximately 3%) had remained attached to the ring or the filters themselves, and few were lost in the glass wool filter. Cell clumps were encountered rarely in the chamber fluid, thereby enabling the use of the glass wool filter without gross cell loss.

To check for possible migration of cells through the filters, 6 normal mice received empty chambers intraperitoneally. Four days later, these mice were inoculated with 0·2 ml. of undiluted fresh tumor fluid. At 10–14 days, when these mice were grossly distended with EAT, they were killed and the chambers examined. These chambers were filled with peritoneal fluid, but there were no cells (EAT, or normal WBC's) seen microscopically, indicating no, or at least very minimal, passage of EAT through the filters from an area of massive tumor concentration in the peritoneal cavity to the inside of the chamber. Animals followed for 60–90 days after EAT-chamber placement did not develop the tumor, even when the chambers remained intraperitoneally, indicating no, or minimal, migration from within a chamber out into the peritoneal cavity.

### DISCUSSION

The method of study used here necessarily influences the growth of intraperitoneal EAT by circumstances non-operative in the absence of millipore chambers. This investigation was specifically aimed at the evaluation of EAT proliferation inside millipore chambers, a circumstance combining an *in vivo* environment with an *in vitro* accounting of total cells. The derived growth curves show exponential growth of EAT essentially without a lag phase, followed by a leveling period. Capalbo, Albright and Bennett (1964) have shown that small numbers of white blood cells can apparently pass through chambers with a 0·45 $\mu$ pore size. The passage of EAT cells did not appear to be a factor in this study, since reverse passage into empty chambers could not be documented, nor did mice with EAT in chambers develop tumors on prolonged follow-up.

Quantitative approaches to ascites tumor growth in mice previously reported include those of Patt and Blackford (1954), Klein and Révész (1953), and Baserga (1963). Many of these studies deal with the estimation of the number of free tumor cells in the peritoneal cavity. The growth curves described resemble those obtained in the present study, with exponential growth followed by a leveling phase. EAT cells in tissue culture also show a similar phasic proliferation.

The growth of EAT in passively immunized mice has been investigated by several groups, including Lindner (1958, 1960) and Levi *et al.* (1959), and such studies demonstrate immune inhibition (Amos and Wakefield, 1958). Also, Harveit (1963, 1962), who has done considerable work with the mouse-EAT system, has shown a cytotoxic factor in the ascitic fluid and has also demonstrated production of active immunization to EAT in mice. However, quantitative studies in actively immunized mice have been largely bypassed, perhaps due to the heterogeneity of the immune response in a group of mice.

The growth of EAT within millipore chambers in actively immunized mice demonstrates inhibition of EAT growth. The EAT cells were not completely destroyed however, but apparently survived at a reduced rate of growth. In those animals that were immunized and later challenged by EAT, immunity was significant but not complete. Variation was noted in fact, regarding tumor challenge in both the normal and immunized mice.

The nature of this mechanism has not been identified by this study. Considerations include the possible transfer of: (a) classic antibody with or without complement, (b) the immune lymphocyte factor of Lawrence *et al.* (1963), (c) immune lymphocyte RNA (Mannick and Egdahl, 1964), (d) other factors to affect EAT cells either directly or in conjunction with trapped leukocytes, and (e) mononuclear cells, though probably in insufficient quantity in this study to achieve the result appreciated by themselves.

### SUMMARY

This investigation documents the quantitative growth of EAT cells in normal and actively immunized mice, using an *in vivo* millipore chamber technique. The presence of an inhibitor of EAT cell growth was demonstrated in actively immunized mice.

This work was supported by Damon Runyon Grants No. 868 and No. 906.

### REFERENCES

AMOS, D. B. AND WAKEFIELD, J.—(1958) *J. natn. Cancer Inst.*, **21**, 657.

BASERGA, R.—(1963) *Archs Path.*, **75**, 58.

CAPALBO, E. E., ALBRIGHT, J. F. AND BENNETT, W. E.—(1964) *J. Immun.*, **92**, 243.

HARVEIT, F.—(1963) *Acta path. microbiol. scand.*, **58**, 10.—(1962) *Br. J. Cancer*, **16**, 331.

KLEIN, G. AND RÉVÉSZ, L.—(1953) *J. natn. Cancer Inst.*, **14**, 229.

LAWRENCE, H. S., AL-ASKARI, S., DAVID, J., FRANKLIN, E. C. AND ZWEIMAN, S.—(1963) *Trans. Ass. Am. Physns*, **76**, 84.

LEVI, E., SCHRECTMA, A., SHERINS, R. AND TOBIAS, S.—(1959) *Nature, Lond.*, **184**, 563.

LINDNER, A.—(1958) *Fedn Proc. Fedn Am. Socs exp. Biol.*, **17**, 446.—(1960) *Am. J. clin. Path.*, **34**, 426.

MANNICK, J. AND EGDAHL, R.—(1964) *J. clin. Invest.*, **43**, 11.

PATT, H. AND BLACKFORD, M.—(1954) *Cancer Res.*, **14**, 391.


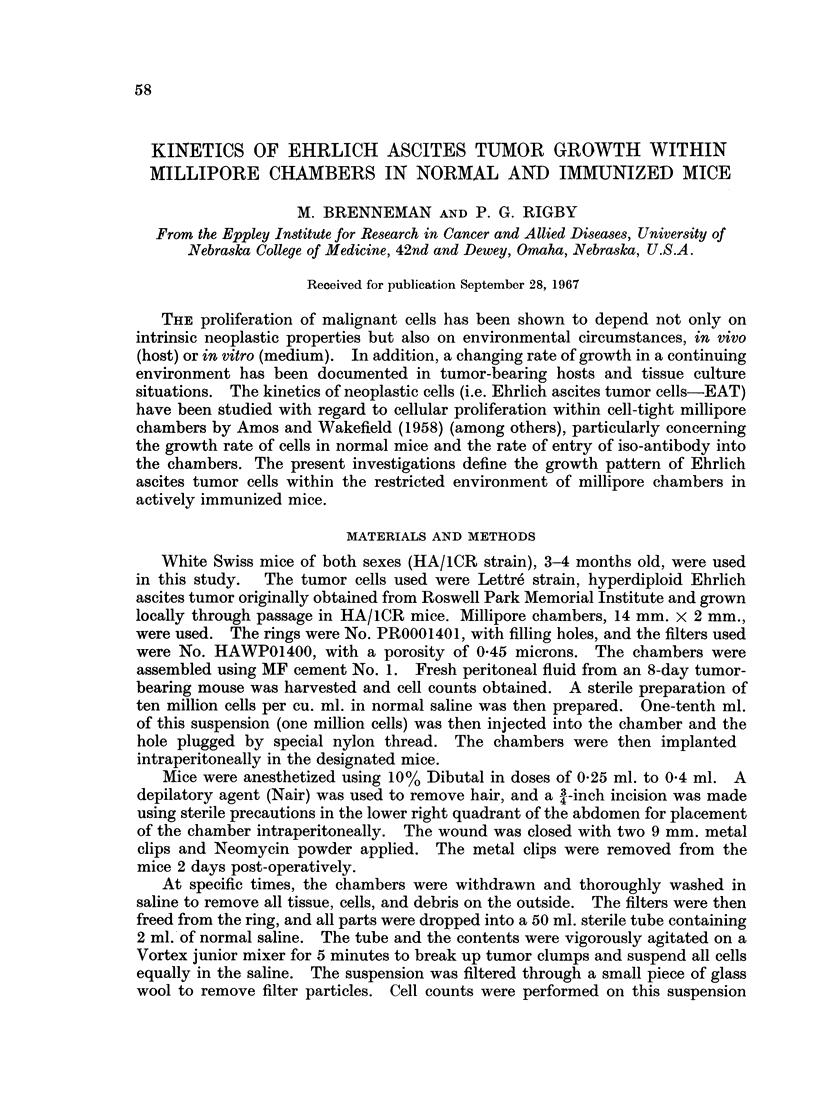

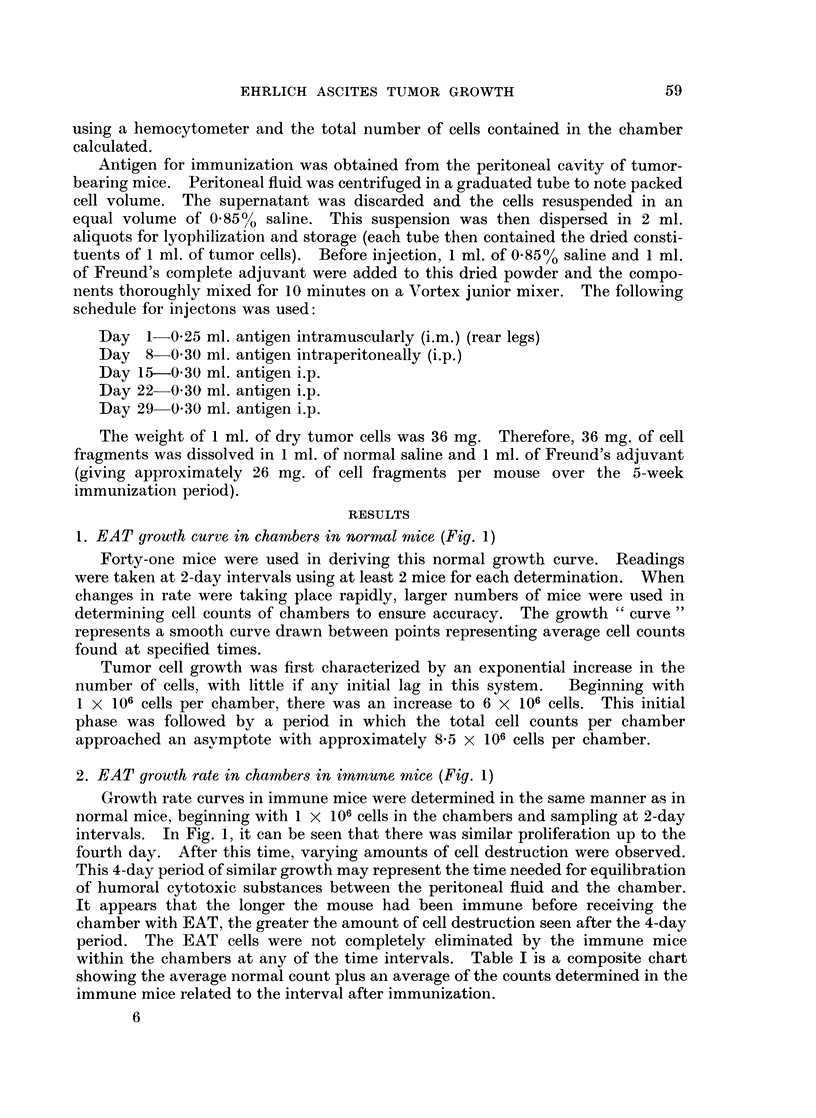

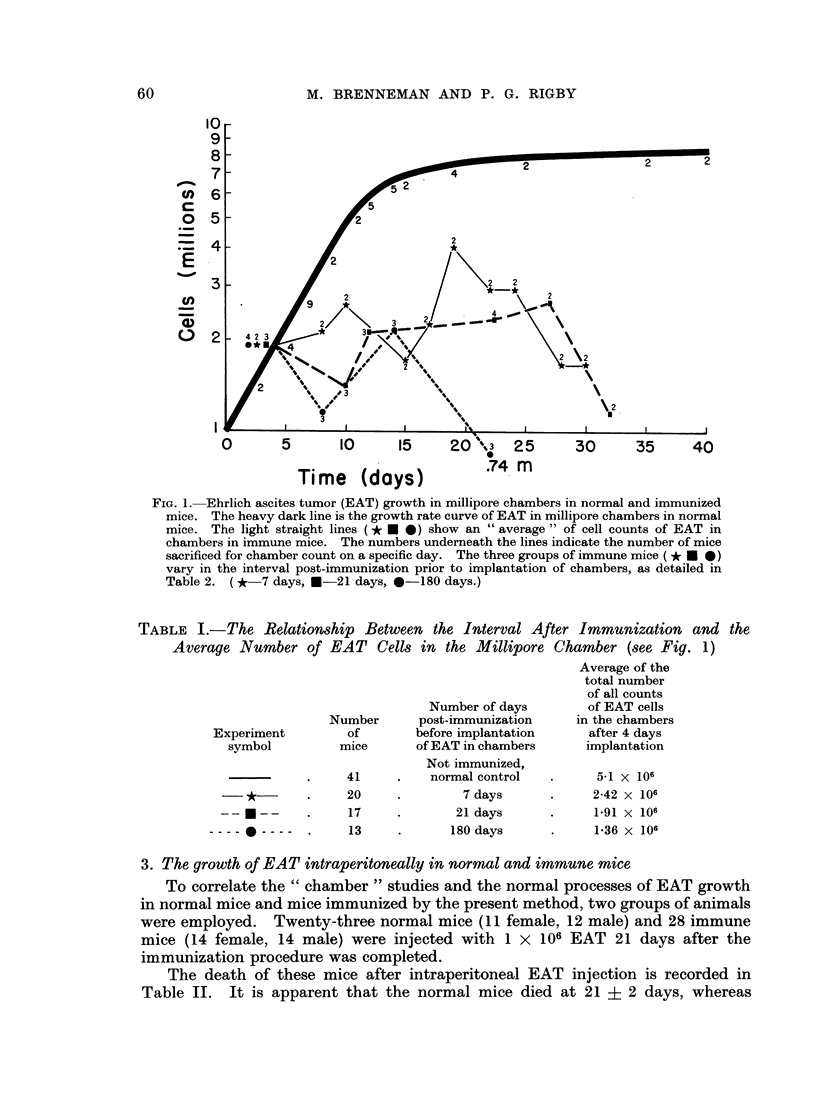

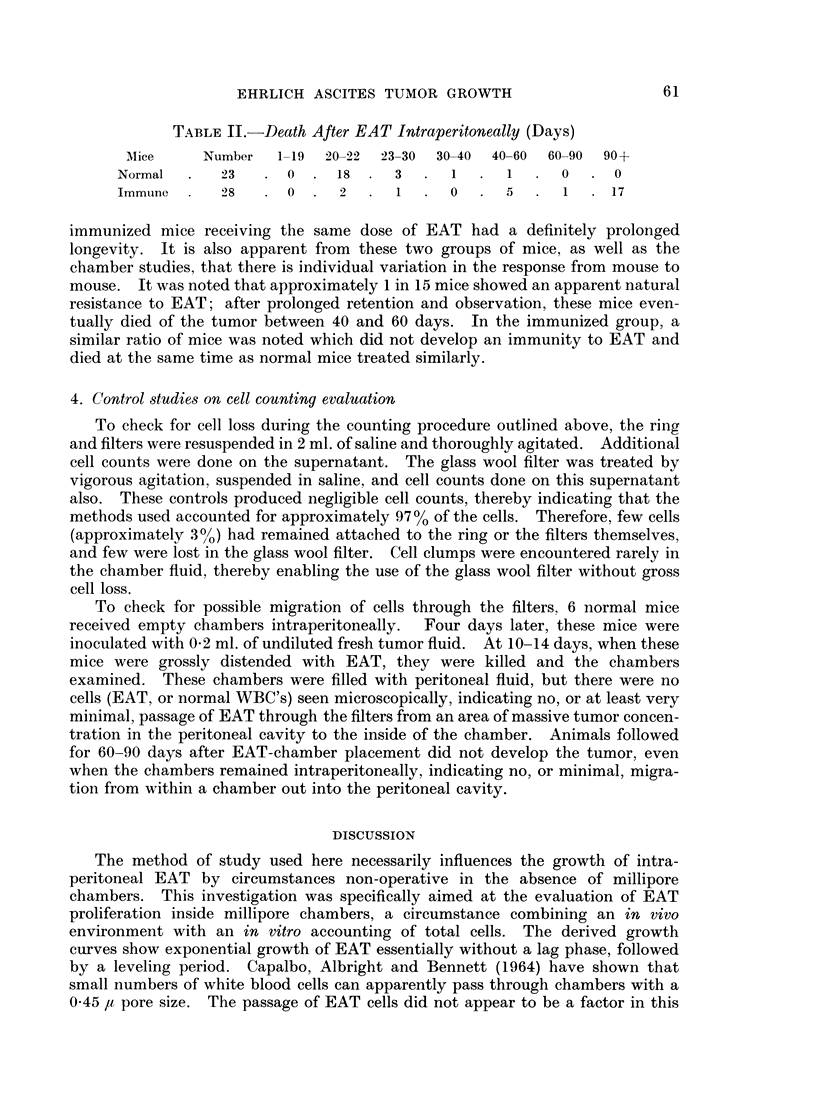

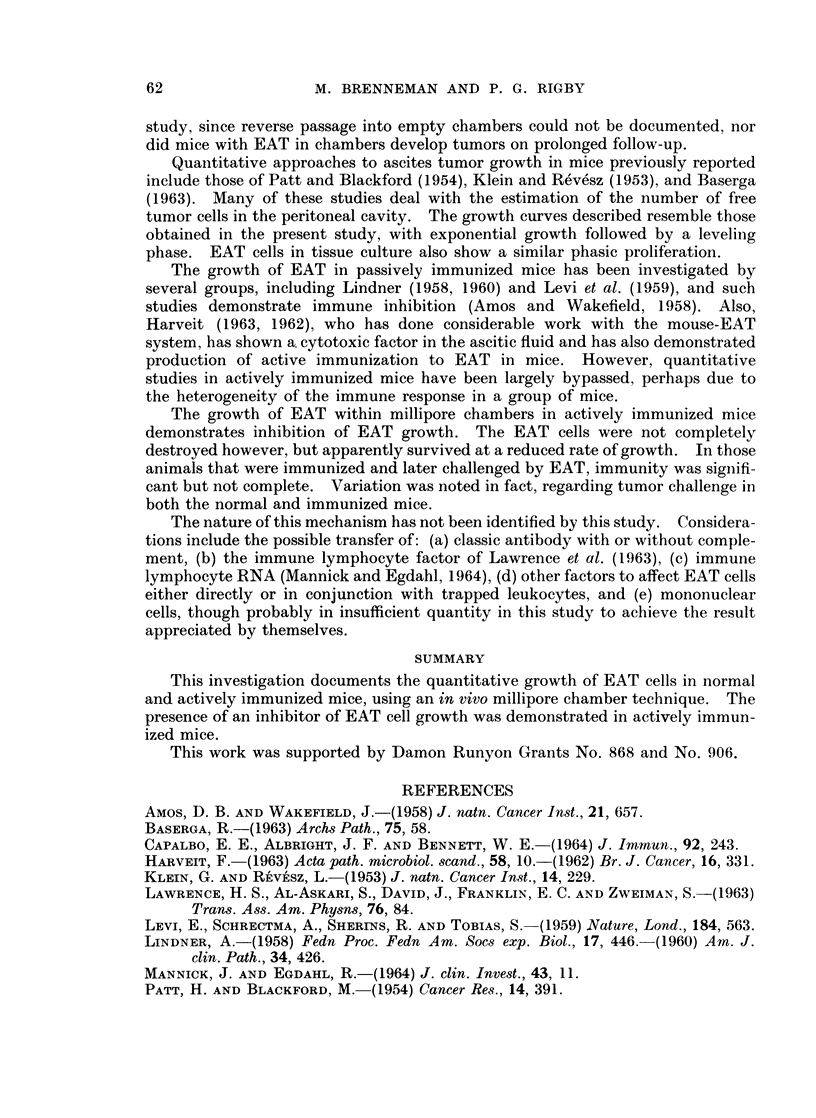


## References

[OCR_00306] AMOS D. B., WAKEFIELD J. D. (1958). Growth of mouse ascites tumor cells in diffusion chambers. I. Studies of growth rate of cells and of the rate of entry of antibody.. J Natl Cancer Inst.

[OCR_00308] CAPALBO E. E., ALBRIGHT J. F., BENNETT W. E. (1964). EVALUATION OF THE DIFFUSION CHAMBER CULTURE TECHNIQUE FOR STUDY OF THE MORPHOLOGICAL AND FUNCTIONAL CHARACTERISTICS OF LYMPHOID CELLS DURING ANTIBODY PRODUCTION.. J Immunol.

[OCR_00311] KLEIN G., REVESZ L. (1953). Quantitative studies on the multiplication of neoplastic cells in vivo. I. Growth curves of the Ehrlich and MC1M ascites tumors.. J Natl Cancer Inst.

[OCR_00317] LEVI E., SCHECHTMAN A. M., SHERINS R. S., TOBIAS S. (1959). Tumour specificity and immunological suppression.. Nature.

[OCR_00318] LINDNER A. (1960). Mechanisms of immune lysis of Ehrlich ascites tumor cells.. Am J Clin Pathol.

[OCR_00323] PATT H. M., BLACKFORD M. E. (1954). Quantitative studies of the growth response of the Krebs ascites tumor.. Cancer Res.

